# Treatment with a Nitric Oxide-Donating NSAID Alleviates Functional Muscle Ischemia in the Mouse Model of Duchenne Muscular Dystrophy

**DOI:** 10.1371/journal.pone.0049350

**Published:** 2012-11-05

**Authors:** Gail D. Thomas, Jianfeng Ye, Claudio De Nardi, Angela Monopoli, Ennio Ongini, Ronald G. Victor

**Affiliations:** 1 The Heart Institute, Cedars-Sinai Medical Center, Los Angeles, California, United States of America; 2 The University of Texas Southwestern Medical Center, Dallas, Texas, United States of America; 3 NicOx Research Institute, Bresso, Italy; University of Minnesota Medical School, United States of America

## Abstract

In patients with Duchenne muscular dystrophy (DMD) and the standard mdx mouse model of DMD, dystrophin deficiency causes loss of neuronal nitric oxide synthase (nNOSμ) from the sarcolemma, producing functional ischemia when the muscles are exercised. We asked if functional muscle ischemia would be eliminated and normal blood flow regulation restored by treatment with an exogenous nitric oxide (NO)-donating drug. Beginning at 8 weeks of age, mdx mice were fed a standard diet supplemented with 1% soybean oil alone or in combination with a low (15 mg/kg) or high (45 mg/kg) dose of HCT 1026, a NO-donating nonsteroidal anti-inflammatory agent which has previously been shown to slow disease progression in the mdx model. After 1 month of treatment, vasoconstrictor responses to intra-arterial norepinephrine (NE) were compared in resting and contracting hindlimbs. In untreated mdx mice, the usual effect of muscle contraction to attenuate NE-mediated vasoconstriction was impaired, resulting in functional ischemia: NE evoked similar decreases in femoral blood flow velocity and femoral vascular conductance (FVC) in the contracting compared to resting hindlimbs (ΔFVC contraction/ΔFVC rest = 0.88±0.03). NE-induced functional ischemia was unaffected by low dose HCT 1026 (ΔFVC ratio = 0.92±0.04; P>0.05 vs untreated), but was alleviated by the high dose of the drug (ΔFVC ratio = 0.22±0.03; P<0.05 vs untreated or low dose). The beneficial effect of high dose HCT 1026 was maintained with treatment up to 3 months. The effect of the NO-donating drug HCT 1026 to normalize blood flow regulation in contracting mdx mouse hindlimb muscles suggests a putative novel treatment for DMD. Further translational research is warranted.

## Introduction

Mutations in the gene encoding the cytoskeletal protein dystrophin cause the X-linked fatal muscle wasting disease Duchenne muscular dystrophy (DMD) [1], [2]. Effective treatment for DMD is lacking, resulting in premature death often before the age of 30 due to respiratory muscle weakness and cardiomyopathy [3], [4]. Both patient survival and quality of life have been improved by home ventilation and corticosteroids, which can prolong ambulation by 2–3 years, reduce the risk of scoliosis, and mitigate pulmonary and cardiac decline in the second decade [3], [4]. Yet corticosteroids are no panacea and cause much toxicity; more than 25% of boys with DMD are not treated due to intolerable side-effects and lack of response [3], [4]. There is an urgent unmet need for effective therapeutic options for this devastating disease.

Dystrophin is a large sub-sarcolemmal protein that provides a physical link between the intracellular actin cytoskeleton and the extracellular matrix [5]. In DMD, loss of dystrophin destabilizes the sarcolemma, rendering the muscle fibers susceptible to physical damage with repeated contraction [6]. Dystrophin also forms a transmembrane complex that targets other proteins to the sarcolemma. Among these proteins is the muscle-specific splice variant of neuronal nitric oxide synthase μ (nNOSμ), which binds spectrin-like repeats within dystrophin's rod domain and the adaptor protein α-syntrophin [7]–[9]. Dystrophin deficiency causes a secondary deficiency of nNOSμ, which is both reduced in content and misplaced from the sarcolemma to the cytosol [7], [8]. Our previous studies in mouse models and patients with DMD have shown that loss of sarcolemmal nNOSμ renders the dystrophic muscle fibers susceptible to functional ischemia and implicated muscle-derived nitric oxide (NO) as a novel therapeutic target for DMD [9]–[12]. Specifically, we found that muscle-derived NO normally attenuates α-adrenergic vasoconstriction in exercising muscle, a protective mechanism termed functional sympatholysis that optimizes muscle blood flow [10]–[14]. This protective mechanism is defective in mdx mice, nNOS null mice, and boys with DMD causing functional muscle ischemia [10], [11]. Repeated ischemic insults may promote fatigue and accelerate use-dependent injury of vulnerable dystrophin-deficient muscles [15], [16].

We recently showed that functional muscle ischemia is prevented in mdx mice by transgenic expression of a dystrophin mini-gene that restores sarcolemmal targeting of nNOSμ, but is unaffected by a dystrophin mini-gene that cannot restore sarcolemmal nNOSμ [9]. That work implicated a pivotal role for endogenous muscle-derived NO to modulate blood flow in contracting mdx skeletal muscle. Because gene therapy for DMD is in an early stage of clinical research, we asked if a simple pharmacological approach that delivers exogenous NO would replicate the dramatic effects of the previous transgenic rescue experiments.

Treatment of mdx mice for six months with HCT 1026, a NO-donating derivative of the nonsteroidal anti-inflammatory drug (NSAID) flurbiprofen, has been shown to slow disease progression by reducing muscle necrosis and inflammation and improving muscle regeneration [17]. Whether improved muscle blood flow regulation contributes to the improved mdx phenotype has not been investigated. Therefore, the aim of this study was to determine if exogenous NO delivery with HCT 1026 would prevent functional muscle ischemia in mdx mice. We performed a short-term treatment study to determine if: (a) muscle blood flow regulation would be restored sooner than the time needed to improve muscle histopathology, and (b) the hemodynamic benefit would persist without development of tolerance as is commonly observed with classical nitrovasodilators.

## Materials and Methods

### Mice and drug

All procedures involving mice were performed in accordance with the US National Institutes of Health Guide for the Care and Use of Laboratory Animals and were approved by the Institutional Animal Care and Use Committee at the University of Texas Southwestern Medical Center (AWA Assurance #A3472-01).

Four- to five-week-old male mdx mice (C57BL/10ScSn-Dmd^mdx^/J; The Jackson Laboratory, Bar Harbor, ME) were housed 3–4 per cage with a 12-h light-dark cycle. Mice were provided a standard rodent diet (7013, NIH-31 Modified 6% Mouse/Rat Diet, Harlan Teklad, Madison, WI) and water ad libitum. Beginning at 8 weeks of age, mice were randomized to receive the standard diet supplemented with 1% soybean oil alone or in combination with HCT 1026 (nitroflurbiprofen; [1,1′-biphenyl]-4-acetic acid-2-fluoro-α-methyl-4-(nitroxy)butyl ester; NicOx, Bresso, Italy) incorporated at concentrations of 100 or 300 parts per million (ppm). These drug concentrations were estimated to provide respective daily doses of approximately 15 or 45 mg of HCT 1026 per kg body weight based on an average daily food consumption of 4–5 g per mouse as determined in preliminary experiments. Mice and food were weighed weekly in order to calculate average daily food consumption and estimate drug doses. Mice were used for hindlimb blood flow experiments after 1, 2, or 3 months of drug treatment.

### Hindlimb blood flow experiment

Mice were anesthetized with a combination of Telazol and xylazine (7.5 and 20 mg/kg, ip). Supplemental doses were administered as needed to maintain anesthesia for the duration of the experiment. Core temperature was monitored using a rectal probe and was maintained at 37°C with an external heat source. A cannula was placed in the trachea to maintain a patent airway and deliver supplemental oxygen by passing a stream of 100% O_2_ mixed with room air across the cannula opening. Catheters were placed in a jugular vein to administer fluids and in a carotid artery to measure arterial blood pressure via a Statham transducer (P23XL). A cuff-type pulsed Doppler flow probe was placed around the left femoral artery to measure blood flow velocity (Crystal Biotech, Matec Instrument Co). Stimulating electrodes were affixed to the left sciatic nerve and were used to evoke hindlimb muscle contractions. The left hindlimb was fixed at the knee joint and connected to a force transducer (FT-10, Grass Instruments) via the calcaneal tendon. A catheter was placed in the right femoral artery and advanced to the aorta and was used to deliver drugs to the left hindlimb. Arterial blood pressure, heart rate (HR), femoral blood flow velocity, and calculated femoral vascular conductance (FVC = mean blood flow velocity/mean arterial pressure) were continuously monitored and digitally recorded at 100 Hz (PowerLab, ADInstruments).

After a 30-minute stabilization period, the vasoconstrictor responses to intra-arterial injections of the α-adrenergic receptor agonist norepinephrine (NE; 2–20 ng in a volume of 2–16 µL) were recorded with the left hindlimb at rest and during intermittent, tetanic contractions produced by stimulation of the sciatic nerve (100-ms trains of pulses [100 Hz, 0.2-ms] at a rate of 30 trains/min) at 2–3 times the motor threshold voltage. At the end of the experiment, mice were euthanized by anesthetic overdose followed by pneumothorax.

### Plasma measurements

Additional mice fed the standard or medicated diet (HCT 1026–300 ppm) for 1 month were anesthetized with isoflurane (2% inhalation) and blood was obtained by direct cardiac puncture. Plasma samples were used for the measurement of flurbiprofen levels by LC-MS/MS (NicOx Research Institute, Bresso, Italy) and creatine kinase activity (Pointe Scientific, Canton, MI).

### Data analysis and statistics

Mean arterial pressure (MAP), HR, femoral blood flow velocity and FVC were measured at rest and during steady-state unilateral hindlimb contractions by averaging 60 seconds of data immediately prior to NE injections. FVC responses to NE were calculated by integrating the area under the response curve (AUC).

Data are presented as mean±SE. Statistical analysis was performed using GraphPad Prism (version 5.0). Statistical significance for multiple group comparisons were determined by ANOVA followed by Bonferroni's multiple comparison test. Within group comparisons were determined by repeated-measures ANOVA followed by Bonferroni's multiple comparison test or by Student's paired *t* test, as appropriate. *P*<0.05 was considered significant.

## Results

### Weight gain and food intake are unaffected by HCT 1026 treatment

Beginning at 8 weeks of age, mdx mice were fed a diet containing either 100 ppm HCT 1026 (n = 29) or 300 ppm HCT 1026 (n = 31) with the goal of attaining daily drug doses of 15 and 45 mg/kg body weight, respectively. Control mice were fed the same diet without any drug (n = 44). HCT 1026 was well tolerated in mice treated with the drug for up to 3 months. Increases in body weight were similar in groups of mice treated for 1, 2, or 3 months with either dose of HCT 1026 compared with untreated mice (Figure S1). Food intake also was similar among treated and untreated groups, remaining relatively constant over the course of the study (Figure S1). Targeted drug doses were achieved in all of the HCT 1026-treated mice: calculated average daily doses based on food intake and body weight were within 9% and 8% of the targeted doses in mice fed the 100 ppm and 300 ppm HCT 1026 diets, respectively (Table 1).

**Table 1:** Drug doses.

	Estimated drug dose (mg/kg/day)		
Treatment	1 month	2 month	3 month
HCT 1026–100 ppm	14.8±0.6	14.6±0.3	13.6±0.1
HCT 1026–300 ppm	43.4±3.7	43.8±1.6	41.5±0.9

Data are mean±SE. Estimates were derived from weekly measurements of body weights and food intake standardized by the number of mice per cage.

### Short-term HCT 1026 alleviates functional muscle ischemia in a dose-dependent manner

We previously showed that endogenous NO produced by sarcolemmal nNOSμ normally opposes an otherwise adverse α-adrenergic vasoconstriction in contracting skeletal muscle and that this protective mechanism is greatly impaired in the mdx mouse due to loss of sarcolemmal nNOSμ [11], [12]. To determine if exogenous NO delivery with HCT 1026 would rescue the blood flow phenotype in the dystrophin-deficient mdx mouse, we evaluated the vasoconstrictor responses to intra-arterial hindlimb infusion of the α-adrenergic receptor agonist NE at rest and during unilateral hindlimb contractions. At rest, MAP, HR, femoral blood flow velocity, and FVC were similar in mice fed the control diet compared to mice fed the 100 ppm or 300 ppm HCT 1026 diet (n = 12 per group) for 1 month (Table 2). The hemodynamic response to unilateral hindlimb contraction alone also was comparable among the three groups of mice: MAP decreased by 5–7% and femoral blood flow velocity and vascular conductance increased by 55–65% and 70–80%, respectively (Table 2). The peak force produced by the contracting hindlimbs was similar in all of the mice (Table 2).

**Table 2:** Hemodynamics and hindlimb force after 1 month of treatment.

	Untreated		HCT 1026–100 ppm		HCT 1026–300 ppm	
	Rest	Contraction	Rest	Contraction	Rest	Contraction
MAP, *mmHg*	103±5	98±6^†^	104±3	98±3^†^	96±3	89±3^†^
HR, *bpm*	457±20	457±25	422±15	455±11^†^	403±19	405±21
Flow, *kHz*	1.1±0.2	1.7±0.3^†^	1.7±0.2	2.8±0.3*^†^	1.6±0.3	2.5±0.4^†^
Conductance, *kHz/mmHg*	0.010±0.002	0.017±0.002^†^	0.016±0.002	0.029±0.004*^†^	0.017±0.003	0.029±0.005*^†^
Peak force, *g*	—	196±6	—	210±13	—	205±11

Data are mean±SE. For each group, n = 12. *P<0.05 vs. untreated; ^†^ *P*<0.05 vs. rest. MAP, mean arterial pressure; HR, heart rate; Flow, femoral blood flow velocity; Conductance, femoral vascular conductance.

**Figure 1:**
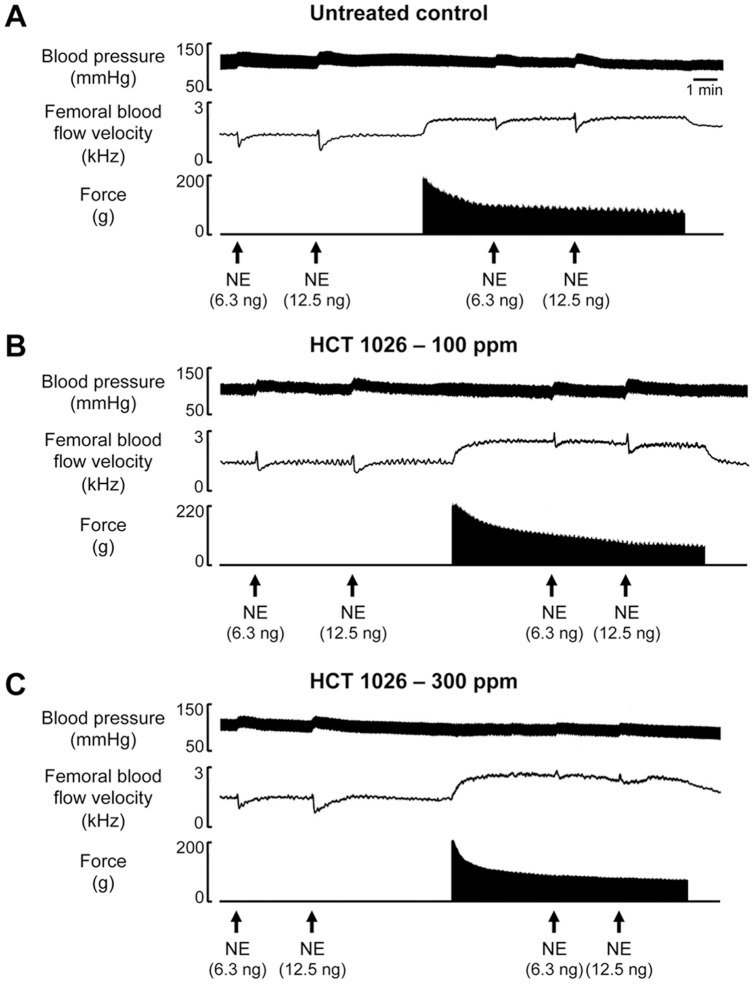
Representative recordings of vasoconstrictor responses to norepinephrine in resting and contracting hindlimbs of mdx mice. (A) Original recording showing that the dose-dependent increases in blood pressure and decreases in femoral blood flow velocity evoked by norepinephrine (NE) in the resting hindlimb were not attenuated in the contracting hindlimb of an untreated mdx mouse, indicating functional muscle ischemia. (B and C) NE-mediated muscle ischemia persisted in the contracting hindlimb of a mouse fed the 100 ppm HCT 1026 diet for 1 month, but was markedly attenuated in the contracting hindlimb of a mouse fed the 300 ppm HCT 1026 diet.

**Figure 2:**
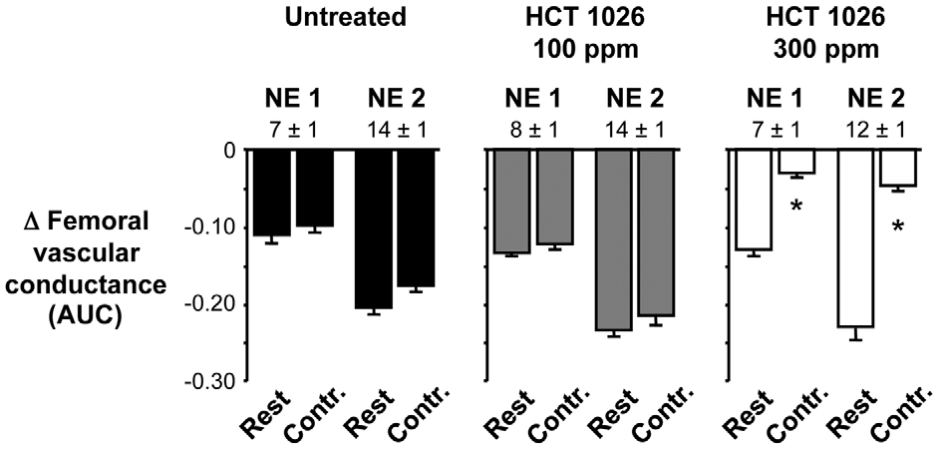
Summary data showing dose-dependent effects of 1-month HCT 1026 treatment on functional muscle ischemia. Graded doses of NE (NE 1, NE 2) evoked decreases in femoral vascular conductance in the resting hindlimbs that were similar among the three groups of mice. These vasoconstrictor responses were attenuated only in the contracting hindlimbs of mice fed the 300 ppm HCT 1026 diet. Numbers above the bars are NE doses in ng. AUC, area under the curve in arbitrary units; Contr., contraction. For each group, n = 12. *P<0.05 vs. corresponding rest.

Injection of graded doses of NE into the resting hindlimbs evoked graded increases in MAP and decreases in femoral blood flow velocity and vascular conductance that were comparable in HCT 1026-treated and untreated mice (Figures 1 and 2). As expected based on our previous studies of mdx mice [9], [11], [12], these vasoconstrictor responses were abnormally preserved in the contracting hindlimbs of the untreated mice: when NE was injected into the contracting hindlimbs, it caused decreases in femoral blood flow velocity and vascular conductance that were similar to the responses observed in the resting hindlimbs (ΔFVC at rest vs. contraction: NE dose 1, −0.110±0.011 vs. −0.097±0.010 AUC, *P*>0.05; NE dose 2, −0.204±0.010 vs. −0.175±0.009 AUC, *P*>0.05) (Figures 1 and 2). This functional muscle ischemia persisted in the mice treated with 100 ppm HCT 1026 (ΔFVC at rest vs. contraction: NE dose 1, −0.134±0.004 vs. −0.123±0.006 AUC, *P*>0.05; NE dose 2, −0.234±0.007 vs. −0.215±0.011 AUC, *P*>0.05). In contrast, NE-mediated vasoconstriction was significantly attenuated in the contracting hindlimbs of the mice treated with 300 ppm HCT 1026 (ΔFVC at rest vs. contraction: NE dose 1, −0.130±0.007 vs. −0.031±0.004 AUC, *P*<0.05; NE dose 2, −0.230±0.015 vs. −0.047±0.006 AUC, *P*<0.05). This higher dose of HCT 1026 was very effective, completely restoring the normal ability of muscle contraction to attenuate NE-mediated vasoconstriction to within the range typically observed in wild type mice in our previous studies [11], [12].

Plasma levels of flurbiprofen (the detectable metabolite of HCT 1026) measured in a subset of mice fed the 300 ppm HCT 1026 diet (n = 5) ranged from 23–35 µM. This concentration is within the expected range based on previous studies in mice [17], [18]. Creatine kinase activity also was measured in this subset of HCT 1026-treated mice (460±144 IU/L; n = 5) and was not different from untreated mdx mice (626±319 IU/L; n = 5; *P*>0.05 vs HCT 1026).

### Beneficial effect of HCT 1026 is sustained with treatment up to 3 months

**Figure 3:**
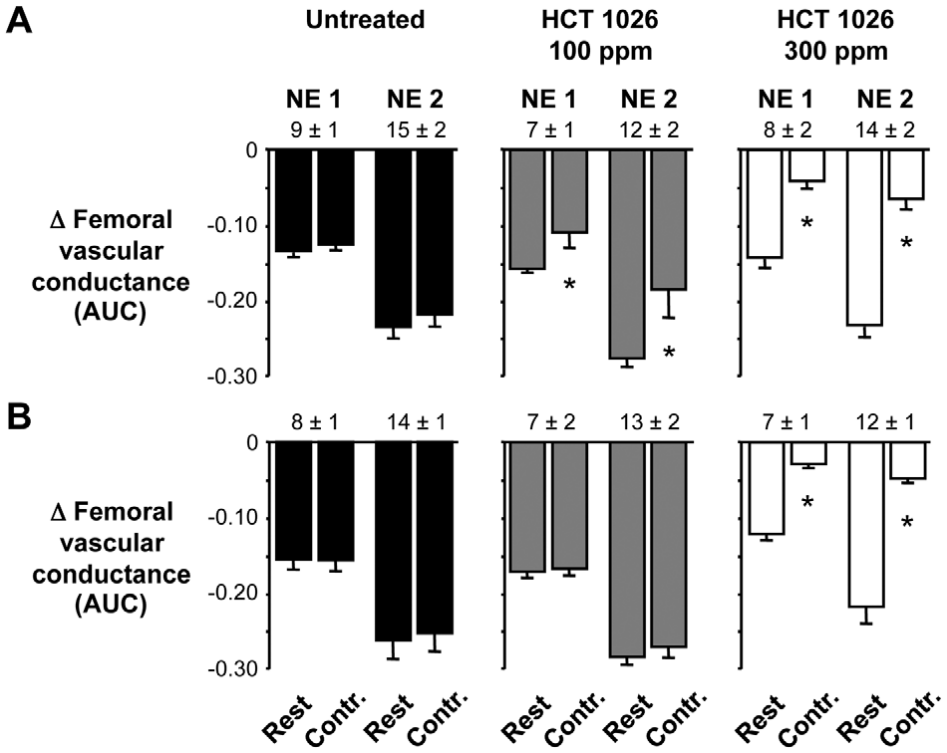
Summary data showing effects of 2- or 3-month HCT 1026 treatment on functional muscle ischemia. (A) In the 2-month treatment groups, the vasoconstrictor responses to graded doses of NE (NE 1, NE 2) were slightly attenuated in the contracting hindlimbs of mice fed the 100 ppm HCT 1026 diet and were greatly attenuated in the contracting hindlimbs of mice fed the 300 ppm HCT 1026 diet. (B) In the 3-month treatment groups, NE-mediated vasoconstriction was only attenuated in the contracting hindlimbs of mice fed the 300 ppm HCT 1026 diet. Numbers above the bars are NE doses in ng. AUC, area under the curve in arbitrary units; Contr., contraction. Untreated, n = 10–11; HCT 100 ppm, n = 8; HCT 300 ppm, n = 5–6. *P<0.05 vs. corresponding rest.

The clinical utility of some NO-donating drugs, such as the organic nitrates, is limited because tolerance to the drugs occurs with chronic administration [19]. To determine if the beneficial effect of HCT 1026 to ameliorate functional muscle ischemia would be sustained during an extended period of treatment, we studied additional groups of mdx mice fed the control or 100 or 300 ppm HCT 1026 diets for 2 or 3 months. As observed in the 1 month treatment group, longer term treatment with HCT 1026 had no adverse effects on MAP, HR, femoral blood flow velocity or FVC at rest (data not shown). The pattern of the hemodynamic response to hindlimb contraction also was unaffected by longer term treatment with HCT 1026 (data not shown). Vasoconstrictor responses to NE injected into the resting hindlimbs were similar in mice fed the control or 100 or 300 ppm HCT 1026 diets for 2 and 3 months (Figure 3). This NE-mediated vasoconstriction was slightly attenuated in the contracting hindlimbs of mice fed the 100 ppm HCT 1026 diet for 2 months and was markedly attenuated in the contracting hindlimbs of mice fed the 300 ppm HCT 1026 diet for 2 and 3 months (Figure 3).

**Figure 4:**
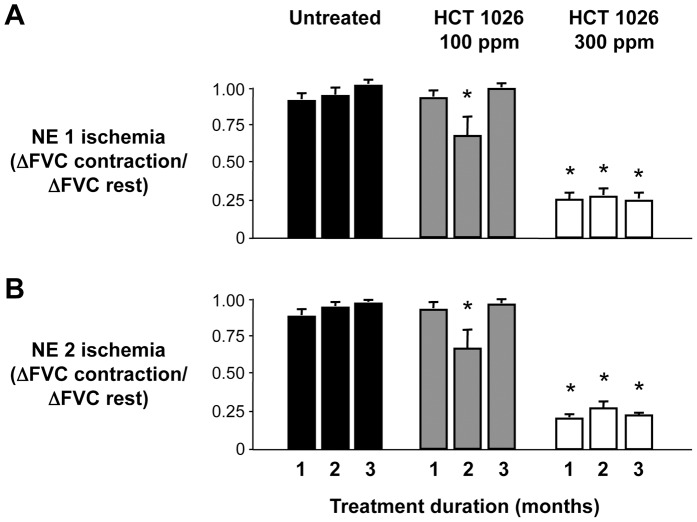
Summary data comparing NE-induced muscle ischemia in untreated and HCT 1026 treated mdx mice. (A) NE (NE 1, 7–9 ng) consistently evoked muscle ischemia in the contracting hindlimbs of untreated mdx mice throughout the 3-month study. Muscle ischemia was only transiently reduced in mice fed the 100 ppm HCT 1026 diet at 2 months, but was prevented in mice fed the 300 ppm HCT 1026 diet for the entire 3-month treatment period. (B) A similar effect of HCT 1026 was observed when a higher dose of NE (NE 2, 12–15 ng) was used to evoke vasoconstriction. Untreated, n = 10–12 mice per group; HCT 100 ppm, n = 8–12 mice per group; HCT 300 ppm, n = 5–12 mice per group. *P<0.05 vs. the same time point in the untreated group.

To directly compare the effect of HCT 1026 to alleviate functional muscle ischemia in the different treatment groups, we derived an ischemic index by calculating the ratio of the FVC responses to NE in contracting versus resting hindlimb where a value of 1 indicates profound ischemia and 0 indicates no ischemia (Figure 4). This ischemic index was close to 1 in mice fed the control diet for 1 month (NE dose 1: 0.90±0.04; NE dose 2: 0.87±0.03) and remained at this high level with continued consumption of the diet for 2 or 3 months. The ischemic index was also high in mice fed the 100 ppm HCT 1026 diet for 1 month (NE dose 1: 0.92±0.04; NE dose 2: 0.92±0.04; *P*>0.05 vs. untreated mice), transiently decreasing after 2 months of treatment (NE dose 1: 0.67±0.12; NE dose 2: 0.66±0.12) but returning to a high level after 3 months of treatment. In marked contrast, the ischemic index was low in mice fed the 300 ppm HCT 1026 diet for 1 month (NE dose 1: 0.25±0.04; NE dose 2: 0.20±0.02; *P*<0.05 vs. untreated mice) and remained at this low level when drug treatment was extended to 2 or 3 months.

## Discussion

We previously showed that loss of sarcolemmal nNOSμ in the dystrophin-deficient muscles of mdx mice and in patients with DMD greatly impairs the normal NO-dependent attenuation of α-adrenergic vasoconstriction in exercising skeletal muscle, causing functional muscle ischemia [9]–[11]. We now show that functional ischemia in mdx mice is dramatically reversed and normal blood flow regulation is fully restored by treatment with the NO-donating drug HCT 1026. The full effect of HCT 1026 was achieved within the first month of treatment and was completely sustained for at least three months without any noticeable adverse systemic effects. These findings suggest that improved muscle blood flow constitutes a putative mechanism by which HCT 1026 slows disease progression in the mdx mouse and further substantiate the promise of NO-donating NSAIDs as a new treatment for DMD [17], [20], [21].

HCT 1026 belongs to a novel class of drug in which a standard cyclooxygenase (COX)-inhibiting NSAID is linked to a NO-donating moiety to produce dual pharmacological action [22], [23]. These COX-inhibiting NO-donating drugs (CINODs) were initially developed as therapeutic alternatives to the parent NSAID for the treatment of osteoarthritis; addition of the NO-donating moiety was intended to mitigate common adverse effects of chronic NSAID use such as gastrointestinal damage and increased blood pressure [24]–[26]. Indeed, we could not test the effect of treatment with flurbiprofen (the parent NSAID) alone in our study because chronic administration causes severe gastrointestinal toxicity [25], [27]. In contrast, the HCT 1026-treated mice in our study exhibited no adverse side effects as evidenced by similar rates of growth and food intake in treated and untreated mice.

Recent preclinical studies suggest that CINODs also have potential to treat muscular dystrophy [17], [21]. Chronic treatment (6–12 months) of mdx mice and α-sarcoglycan null mice with HCT 1026 increased muscle strength and reduced muscle fatigue, resulting in improved locomotor function [17]. Similar effects have been observed in α-sarcoglycan null mice treated with NCX 320, a NO-donating ibuprofen [21]. Of note, a similar pharmacological approach using the co-administration of the NO donor isosorbide dinitrate and ibuprofen was shown to be safe and well-tolerated in a recent 12-month pilot study in adult dystrophic patients [20]. The beneficial effects of CINOD treatment in the dystrophic mice were attributed to reduced necrosis, reduced inflammation, and preserved regenerative capacity of muscle [17], [21]. However, a mechanistic role for muscle blood flow was not addressed in either of these studies.

Our data now implicate a novel vascular mechanism of action of CINODs: elimination of functional muscle ischemia. The effect was both dose-dependent and impressive. When mdx mice were treated with high dose HCT 1026, NE-mediated vasoconstriction in the contracting hindlimbs was attenuated by 75±4%. We have previously observed the same degree of modulation in untreated wild-type C57BL10 mice (75±10%) and in transgenic mdx mice expressing a dystrophin mini-gene that restores sarcolemmal nNOSμ (81±3%) [9], [11]. Thus, simply delivering exogenous NO with HCT 1026 to dystrophin-deficient mdx mice seems to fully reproduce the beneficial effect of the transgenic rescue experiments on muscle blood flow regulation. This is remarkable because the transgenic mice produce a truncated dystrophin that restores sarcolemmal nNOSμ along with the rest of the dystrophin-glycoprotein complex, while the pharmacological approach presumably works by restoring NO without sarcolemmal targeting in the continued absence of the dystrophin complex [9]. Importantly, blood levels of drug achieved with high dose HCT 1026 in our mdx mouse experiments were similar to levels reported in other rodent models [17], [18], [28] as well as in Phase 1 clinical studies [29] NicOx unpublished data].

That the beneficial effect of high dose HCT 1026 to prevent functional muscle ischemia was fully maintained for the entire three month treatment negates the concern that tolerance to the drug might develop with chronic use. This finding differentiates HCT 1026 from other commonly used organic nitrates such as nitroglycerin, which engenders nitrate tolerance due to oxidative modification and subsequent inhibition of the mitochondrial enzyme responsible for biotransformation of nitroglycerin to NO [19]. Indeed, in healthy rats and humans we found that treatment with nitroglycerin for 6 days induced nitrate tolerance and impaired the normal NO-dependent attenuation of skeletal muscle blood flow during exercise, resulting in functional muscle ischemia similar to that observed in the untreated mdx mice in the present study [30].

The mechanism of NO release from HCT 1026 is not completely understood, but is thought to involve rapid cleavage of flurbiprofen from the nitroxybutyl moiety followed by slower conversion of nitroxybutyl to NO [22], [31]. Following oral administration of a single 15 mg/kg dose of HCT 1026 in rats, plasma levels of flurbiprofen and NOx (nitrite and nitrate, the main metabolic products of NO) rise in parallel, peaking at 6 hours post-administration [28]. While we could not test the effect of flurbiprofen alone in this study, our prior work suggests that the dramatic rescue of the blood flow phenotype by HCT 1026 is far more likely due to NO donation than to flurbiprofen-mediated COX inhibition and reduced prostaglandin synthesis [9], [11], [12], [14]. We previously showed that NSAIDs (indomethacin or aspirin) have no effect on α-adrenergic vasoconstriction in the contracting muscles of healthy rats or humans [14], [32]. Moreover, in isolated blood vessels and perfused kidneys preconstricted with NE or epinephrine (both mediated by α-adrenergic receptors), concentrations of HCT 1026 within the range of calculated plasma levels of the high dose-treated mice in our study (35–51 µM) produced vasorelaxation while flurbiprofen was without effect [33], [34].

A distinctive finding of our study is that NE-mediated vasoconstriction was attenuated in the contracting, but not resting, hindlimbs of the HCT 1026-treated mice. HCT 1026 treatment also did not lower blood pressure, which is consistent with previous studies of normotensive animals [27]. These observations argue against a general vasodilatory action of HCT 1026 and suggest a more specific mechanism by which the drug affects vascular regulation only in the active muscles. One such mechanism could involve conversion of NO derived from HCT 1026 to more stable compounds such as nitrosylhemoglobin or NOx that can circulate in the blood and, in the case of NOx, accumulate in tissues [22], [28], [31], [35], [36]. Release of NO from these compounds is greatly facilitated by physiological levels of hypoxia and acidosis, providing a plausible mechanism to deliver NO bioactivity to ischemic tissue [37]–[39]. Thus, our working mechanistic hypothesis is that greater local NO release occurs when tissue pO_2_ and pH decline during muscle contraction than when the muscles are resting. Such regulated NO release constitutes an attractive hypothesis to explain the selective attenuation of NE-mediated vasoconstriction that we observed in the contracting hindlimbs of HCT 1026-treated mice. Furthermore, our data showing a consistent effect of high dose, but not low dose, HCT 1026 to improve muscle blood flow regulation in the mdx mice suggest that a threshold level of NO bioactivity in blood or muscle is required to rescue the blood flow phenotype. As previous rat studies have shown dose-dependent increases in NOx in blood and brain after oral administration of HCT 1026 in doses ranging from 10 to 50 mg/kg, we speculate that in our study NOx was elevated to a greater extent in mice treated with the high versus the low dose of HCT 1026 (≈ 45 vs. 15 mg/kg) [27], [36].

Our study has several limitations. Our findings do not prove that normalized muscle blood flow regulation is a causal mechanism by which HCT 1026 improves muscle function and histopathology in mdx mice [17]. However, the improved blood flow regulation occurred early in treatment compared to the slower time course of disease improvement (1 month vs. 6 months), which is at least consistent with causal attribution. Further research is needed to define the relative contributions of vascular and non-vascular actions of exogenous NO in this setting. This is particularly important because there are both potential advantages and disadvantages of a NO donor as the means to restore NO signaling in diseased muscle. On the positive side, it may correct cGMP-mediated vascular regulation as shown here, but also S-nitrosylation-mediated inhibition of histone deacetylases and regulation of specific microRNAs thereby improving muscle differentiation and regeneration [40], [41]. On the other hand, increasing cytosolic NO may cause hypernitrosylation of ryanodine receptors thus impairing muscle force or activation of Foxo transcription factors thereby accelerating muscle wasting [42]–[44]. Finally, our study was performed in the mdx mouse, which is an imperfect milder model of DMD. Many of the therapeutic strategies that have shown great promise in the mdx mouse have not translated to benefit patients with DMD. As the CINOD class of NO-donating agents has proven safe in clinical trials [29], [45], translational studies are feasible to determine if these drugs also can alleviate functional muscle ischemia and slow disease progression in boys with DMD.

Despite these limitations, our findings indicate that simply delivering exogenous NO via HCT 1026 to mdx mice, which like DMD patients lack all components of the dystrophin-glycoprotein complex, fully replicates the physiological role of sarcolemmal nNOSμ-derived NO to modulate α-adrenergic vasoconstriction in exercising muscle. Other strategies to improve NO signaling in the mdx mouse currently under investigation include supplementation with the NOS substrate L-arginine to increase NO production and treatment with phosphodiesterase 5A (PDE5A) inhibitors to prolong NO-cGMP signaling [15], [46]–[54]. Those approaches depend on the presence of functional nNOSμ, which is reduced in content and mislocalized from the sarcolemma in dystrophic muscle [7]–[9], [52], [55]. Conceivably, some muscular dystrophy patients may have insufficient nNOSμ for these approaches to be effective. For such patients, NO-donating drugs like HCT 1026 that circumvent the inadequate endogenous NO production may provide benefit as monotherapy or as low dose combination therapy with L-arginine or PDE5A inhibitors to maximize efficacy while minimizing dose-dependent side effects. Further translational and clinical research is warranted.

## Supporting Information

Figure S1 **Effect of HCT 1026 treatment on growth and food intake.** HCT 1026 was well tolerated by mdx mice with no adverse effects on (A) growth or (B) food intake in treated compared to untreated groups. One-month treatment, n = 13–17 mice per group; two-month treatment, n = 7–15 mice per group; three-month treatment, n = 7–14 mice per group.
(PDF)

## References

[1] Koenig M, Hoffman EP, Bertelson CJ, Monaco AP, Feener C, et al (1987) Complete cloning of the Duchenne muscular dystrophy (DMD) cDNA and preliminary genomic organization of the DMD gene in normal and affected individuals. Cell 50: 509–517. 3607877 10.1016/0092-8674(87)90504-6

[2] Monaco AP, Neve RL, Colletti-Feener C, Bertelson CJ, Kurnit DM, et al (1986) Isolation of candidate cDNAs for portions of the Duchenne muscular dystrophy gene. Nature 323: 646–650. 3773991 10.1038/323646a0

[3] Bushby K, Finkel R, Birnkrant DJ, Case LE, Clemens PR, et al (2010) Diagnosis and management of Duchenne muscular dystrophy, part 2: implementation of multidisciplinary care. Lancet Neurol 9: 177–189. 19945914 10.1016/S1474-4422(09)70272-8

[4] Bushby K, Finkel R, Birnkrant DJ, Case LE, Clemens PR, et al (2010) Diagnosis and management of Duchenne muscular dystrophy, part 1: diagnosis, and pharmacological and psychosocial management. Lancet Neurol 9: 77–93. 19945913 10.1016/S1474-4422(09)70271-6

[5] Blake DJ, Weir A, Newey SE, Davies KE (2002) Function and genetics of dystrophin and dystrophin-related proteins in muscle. Physiol Rev 82: 291–329. 11917091 10.1152/physrev.00028.2001

[6] Petrof BJ, Shrager JB, Stedman HH, Kelly AM, Sweeney HL (1993) Dystrophin protects the sarcolemma from stresses developed during muscle contraction. Proc Natl Acad Sci U S A 90: 3710–3714. 8475120 10.1073/pnas.90.8.3710 PMC46371

[7] Brenman JE, Chao DS, Xia H, Aldape K, Bredt DS (1995) Nitric oxide synthase complexed with dystrophin and absent from skeletal muscle sarcolemma in Duchenne muscular dystrophy. Cell 82: 743–752. 7545544 10.1016/0092-8674(95)90471-9

[8] Chang WJ, Iannaccone ST, Lau KS, Masters BSS, McCabe TJ, et al (1996) Neuronal nitric oxide synthase and dystrophin-deficient muscular dystrophy. Proc Natl Acad Sci U S A 93: 9142–9147. 8799168 10.1073/pnas.93.17.9142 PMC38609

[9] Lai Y, Thomas GD, Yue Y, Yang HT, Li D, et al (2009) Dystrophins carrying spectrin-like repeats 16/17 anchor nNOS to the sarcolemma and enhance exercise performance. J Clin Invest 119: 624–635. 19229108 10.1172/JCI36612 PMC2648692

[10] Sander M, Chavoshan B, Harris SA, Iannoccone ST, Stull JT, et al (2000) Functional muscle ischemia in neuronal nitric oxide synthase-deficient skeletal muscle of children with Duchenne muscular dystrophy. Proc Natl Acad Sci U S A 97: 13818–13823. 11087833 10.1073/pnas.250379497 PMC17659

[11] Thomas GD, Sander M, Lau KS, Huang PL, Stull JT, et al (1998) Impaired metabolic modulation of α-adrenergic vasoconstriction in dystrophin-deficient skeletal muscle. Proc Natl Acad Sci U S A 95: 15090–15095. 9844020 10.1073/pnas.95.25.15090 PMC24580

[12] Thomas GD, Shaul PW, Yuhanna IS, Froehner SC, Adams ME (2003) Vasomodulation by skeletal muscle-derived nitric oxide requires alpha-syntrophin-mediated sarcolemmal localization of neuronal nitric oxide synthase. Circ Res 92: 554–560. 12600881 10.1161/01.RES.0000061570.83105.52

[13] Chavoshan B, Sander M, Sybert TE, Hansen J, Victor RG, et al (2002) Nitric oxide-dependent modulation of sympathetic neural control of oxygenation in exercising human skeletal muscle. J Physiol 540: 377–386. 11927694 10.1113/jphysiol.2001.013153 PMC2290221

[14] Thomas GD, Victor RG (1998) Nitric oxide mediates contraction-induced attenuation of sympathetic vasoconstriction in rat skeletal muscle. J Physiol 506: 817–826. 9503340 10.1111/j.1469-7793.1998.817bv.x PMC2230749

[15] Asai A, Sahani N, Kaneki M, Ouchi Y, Martyn JA, et al (2007) Primary role of functional ischemia, quantitative evidence for the two-hit mechanism, and phosphodiesterase-5 inhibitor therapy in mouse muscular dystrophy. PLoS ONE 2: e806. 17726536 10.1371/journal.pone.0000806 PMC1950086

[16] Rando TA (2001) Role of nitric oxide in the pathogenesis of muscular dystrophies: a "two hit" hypothesis of the cause of muscle necrosis. Microsc Res Tech 55: 223–235. 11748861 10.1002/jemt.1172

[17] Brunelli S, Sciorati C, D'Antona G, Innocenzi A, Covarello D, et al (2007) Nitric oxide release combined with nonsteroidal antiinflammatory activity prevents muscular dystrophy pathology and enhances stem cell therapy. Proc Natl Acad Sci U S A 104: 264–269. 17182743 10.1073/pnas.0608277104 PMC1765447

[18] Furlan R, Kurne A, Bergami A, Brambilla E, Maucci R, et al (2004) A nitric oxide releasing derivative of flurbiprofen inhibits experimental autoimmune encephalomyelitis. J Neuroimmunol 150: 10–19. 15081244 10.1016/j.jneuroim.2004.01.004

[19] Munzel T, Daiber A, Gori T (2011) Nitrate therapy: new aspects concerning molecular action and tolerance. Circulation 123: 2132–2144. 21576678 10.1161/CIRCULATIONAHA.110.981407

[20] D'Angelo MG, Gandossini S, Martinelli Boneschi F, Sciorati C, Bonato S, et al (2012) Nitric oxide donor and non steroidal anti inflammatory drugs as a therapy for muscular dystrophies: evidence from a safety study with pilot efficacy measures in adult dystrophic patients. Pharmacol Res 65: 472–479. 22306844 10.1016/j.phrs.2012.01.006

[21] Sciorati C, Miglietta D, Buono R, Pisa V, Cattaneo D, et al (2011) A dual acting compound releasing nitric oxide (NO) and ibuprofen, NCX 320, shows significant therapeutic effects in a mouse model of muscular dystrophy. Pharmacol Res 64: 210–217. 21609764 10.1016/j.phrs.2011.05.003 PMC3134707

[22] Keeble JE, Moore PK (2002) Pharmacology and potential therapeutic applications of nitric oxide-releasing non-steroidal anti-inflammatory and related nitric oxide-donating drugs. Br J Pharmacol 137: 295–310. 12237248 10.1038/sj.bjp.0704876 PMC1573498

[23] Wallace JL, Viappiani S, Bolla M (2009) Cyclooxygenase-inhibiting nitric oxide donators for osteoarthritis. Trends Pharmacol Sci 30: 112–117. 19230986 10.1016/j.tips.2009.01.001

[24] Muscara MN, McKnight W, Lovren F, Triggle CR, Cirino G, et al (2000) Antihypertensive properties of a nitric oxide-releasing naproxen derivative in two-kidney, one-clip rats. Am J Physiol Heart Circ Physiol 279: H528–H535. 10924050 10.1152/ajpheart.2000.279.2.H528

[25] Bertrand V, Guimbaud R, Sogni P, Lamrani A, Mauprivez C, et al (1998) Role of tumour necrosis factor-alpha and inducible nitric oxide synthase in the prevention of nitro-flurbiprofen small intestine toxicity. Eur J Pharmacol 356: 245–253. 9774256 10.1016/s0014-2999(98)00550-0

[26] White WB, Schnitzer TJ, Fleming R, Duquesroix B, Beekman M (2009) Effects of the cyclooxygenase inhibiting nitric oxide donator naproxcinod versus naproxen on systemic blood pressure in patients with osteoarthritis. Am J Cardiol 104: 840–845. 19733721 10.1016/j.amjcard.2009.05.014

[27] Maffia P, Ianaro A, Sorrentino R, Lippolis L, Maiello FM, et al (2002) Beneficial effects of NO-releasing derivative of flurbiprofen (HCT-1026) in rat model of vascular injury and restenosis. Arterioscler Thromb Vasc Biol 22: 263–267. 11834526 10.1161/hq0202.104064

[28] Aldini G, Carini M, Orioli M, Facino RM, Wenk GL (2002) Metabolic profile of NO-flurbiprofen (HCT1026) in rat brain and plasma: a LC-MS study. Life Sci 71: 1487–1500. 12127904 10.1016/s0024-3205(02)01915-x

[29] Zacharowski P, Zacharowski K, Donnellan C, Johnston A, Vojnovic I, et al (2004) The effects and metabolic fate of nitroflurbiprofen in healthy volunteers. Clin Pharmacol Ther 76: 350–358. 15470334 10.1016/j.clpt.2004.05.008

[30] Fadel PJ, Farias III M, Gallagher KM, Wang Z, Thomas GD (2012) Oxidative stress and enhanced sympathetic vasoconstriction in contracting muscles of nitrate-tolerant rats and humans. J Physiol 590: 395–407. 22106180 10.1113/jphysiol.2011.218917 PMC3285073

[31] Govoni M, Casagrande S, Maucci R, Chiroli V, Tocchetti P (2006) In vitro metabolism of (nitrooxy)butyl ester nitric oxide-releasing compounds: comparison with glyceryl trinitrate. J Pharmacol Exp Ther 317: 752–761. 16424150 10.1124/jpet.105.097469

[32] Hansen J, Thomas GD, Harris SA, Parsons WJ, Victor RG (1996) Differential sympathetic neural control of oxygenation in resting and exercising human skeletal muscle. J Clin Invest 98: 584–596. 8755671 10.1172/JCI118826 PMC507464

[33] Adami A, Cuzzolin L, Minuz P, Crivellente F, Lechi A, et al (1996) Vasodilating properties of a new non-steroidal anti-inflammatory drug, nitroflurbiprofen, on rat aortic rings. Pharmacol Res 33: 239–244. 8938015 10.1006/phrs.1996.0033

[34] Keeble J, Al-Swayeh OA, Moore PK (2001) Vasorelaxant effect of nitric oxide releasing steroidal and nonsteroidal anti-inflammatory drugs. Br J Pharmacol 133: 1023–1028. 11487511 10.1038/sj.bjp.0704161 PMC1572867

[35] Carini M, Aldini G, Stefani R, Orioli M, Facino RM (2001) Nitrosylhemoglobin, an unequivocal index of nitric oxide release from nitroaspirin: in vitro and in vivo studies in the rat by ESR spectroscopy. J Pharm Biomed Anal 26: 509–518. 11516901 10.1016/s0731-7085(01)00478-2

[36] Prosperi C, Scali C, Pepeu G, Casamenti F (2001) NO-flurbiprofen attenuates excitotoxin-induced brain inflammation, and releases nitric oxide in the brain. Jpn J Pharmacol 86: 230–235. 11459126 10.1254/jjp.86.230

[37] Lundberg JO, Weitzberg E, Gladwin MT (2008) The nitrate-nitrite-nitric oxide pathway in physiology and therapeutics. Nat Rev Drug Discov 7: 156–167. 18167491 10.1038/nrd2466

[38] van Faassen EE, Bahrami S, Feelisch M, Hogg N, Kelm M, et al (2009) Nitrite as regulator of hypoxic signaling in mammalian physiology. Med Res Rev 29: 683–741. 19219851 10.1002/med.20151 PMC2725214

[39] Zweier JL, Li H, Samouilov A, Liu X (2010) Mechanisms of nitrite reduction to nitric oxide in the heart and vessel wall. Nitric Oxide 22: 83–90. 20044016 10.1016/j.niox.2009.12.004 PMC2851168

[40] Cacchiarelli D, Martone J, Girardi E, Cesana M, Incitti T, et al (2010) MicroRNAs involved in molecular circuitries relevant for the Duchenne muscular dystrophy pathogenesis are controlled by the dystrophin/nNOS pathway. Cell Metab 12: 341–351. 20727829 10.1016/j.cmet.2010.07.008

[41] Colussi C, Mozzetta C, Gurtner A, Illi B, Rosati J, et al (2008) HDAC2 blockade by nitric oxide and histone deacetylase inhibitors reveals a common target in Duchenne muscular dystrophy treatment. Proc Natl Acad Sci U S A 105: 19183–19187. 19047631 10.1073/pnas.0805514105 PMC2614736

[42] Bellinger AM, Reiken S, Carlson C, Mongillo M, Liu X, et al (2009) Hypernitrosylated ryanodine receptor calcium release channels are leaky in dystrophic muscle. Nat Med 15: 325–330. 19198614 10.1038/nm.1916 PMC2910579

[43] Li D, Yue Y, Lai Y, Hakim CH, Duan D (2011) Nitrosative stress elicited by nNOSmicro delocalization inhibits muscle force in dystrophin-null mice. J Pathol 223: 88–98. 21125668 10.1002/path.2799 PMC3109084

[44] Suzuki N, Motohashi N, Uezumi A, Fukada S, Yoshimura T, et al (2007) NO production results in suspension-induced muscle atrophy through dislocation of neuronal NOS. J Clin Invest 117: 2468–2476. 17786240 10.1172/JCI30654 PMC1952622

[45] Karlsson J, Pivodic A, Aguirre D, Schnitzer TJ (2009) Efficacy, safety, and tolerability of the cyclooxygenase-inhibiting nitric oxide donator naproxcinod in treating osteoarthritis of the hip or knee. J Rheumatol 36: 1290–1297. 19411388 10.3899/jrheum.081011

[46] Adamo CM, Dai DF, Percival JM, Minami E, Willis MS, et al (2010) Sildenafil reverses cardiac dysfunction in the mdx mouse model of Duchenne muscular dystrophy. Proc Natl Acad Sci U S A 107: 19079–19083. 20956307 10.1073/pnas.1013077107 PMC2973894

[47] Archer JD, Vargas CC, Anderson JE (2006) Persistent and improved functional gain in mdx dystrophic mice after treatment with L-arginine and deflazacort. FASEB J 20: 738–740. 16464957 10.1096/fj.05-4821fje

[48] Barton ER, Morris L, Kawana M, Bish LT, Toursel T (2005) Systemic administration of L-arginine benefits mdx skeletal muscle function. Muscle Nerve 32: 751–760. 16116642 10.1002/mus.20425

[49] Guerron AD, Rawat R, Sali A, Spurney CF, Pistilli E, et al (2010) Functional and molecular effects of arginine butyrate and prednisone on muscle and heart in the mdx mouse model of Duchenne Muscular Dystrophy. PLoS ONE 5: e11220. 20574530 10.1371/journal.pone.0011220 PMC2888587

[50] Hnia K, Gayraud J, Hugon G, Ramonatxo M, De La Porte S, et al (2008) L-arginine decreases inflammation and modulates the nuclear factor-kappaB/matrix metalloproteinase cascade in mdx muscle fibers. Am J Pathol 172: 1509–1519. 18458097 10.2353/ajpath.2008.071009 PMC2408412

[51] Khairallah M, Khairallah RJ, Young ME, Allen BG, Gillis MA, et al (2008) Sildenafil and cardiomyocyte-specific cGMP signaling prevent cardiomyopathic changes associated with dystrophin deficiency. Proc Natl Acad Sci U S A 105: 7028–7033. 18474859 10.1073/pnas.0710595105 PMC2383977

[52] Kobayashi YM, Rader EP, Crawford RW, Iyengar NK, Thedens DR, et al (2008) Sarcolemma-localized nNOS is required to maintain activity after mild exercise. Nature 456: 511–515. 18953332 10.1038/nature07414 PMC2588643

[53] Percival JM, Whitehead NP, Adams ME, Adamo CM, Beavo JA, et al (2012) Sildenafil Reduces Respiratory Muscle Weakness and Fibrosis in the mdx Mouse Model of Duchenne Muscular Dystrophy. J Pathol 228: 77–87. 22653783 10.1002/path.4054 PMC4067455

[54] Voisin V, Sebrie C, Matecki S, Yu H, Gillet B, et al (2005) L-arginine improves dystrophic phenotype in mdx mice. Neurobiol Dis 20: 123–130. 16137573 10.1016/j.nbd.2005.02.010

[55] Finanger Hedderick EL, Simmers JL, Soleimani A, Andres-Mateos E, Marx R, et al (2011) Loss of sarcolemmal nNOS is common in acquired and inherited neuromuscular disorders. Neurology 76: 960–967. 21403107 10.1212/WNL.0b013e31821043c8 PMC3059139

