# Supplementary figures and images for: Treatment with a Nitric Oxide-Donating NSAID Alleviates Functional Muscle Ischemia in the Mouse Model of Duchenne Muscular Dystrophy
Source: PLoS One. 2012 Nov 5;7(11):e49350. doi: 10.1371/journal.pone.0049350 (PMC3489726; doi:10.1371/journal.pone.0049350)

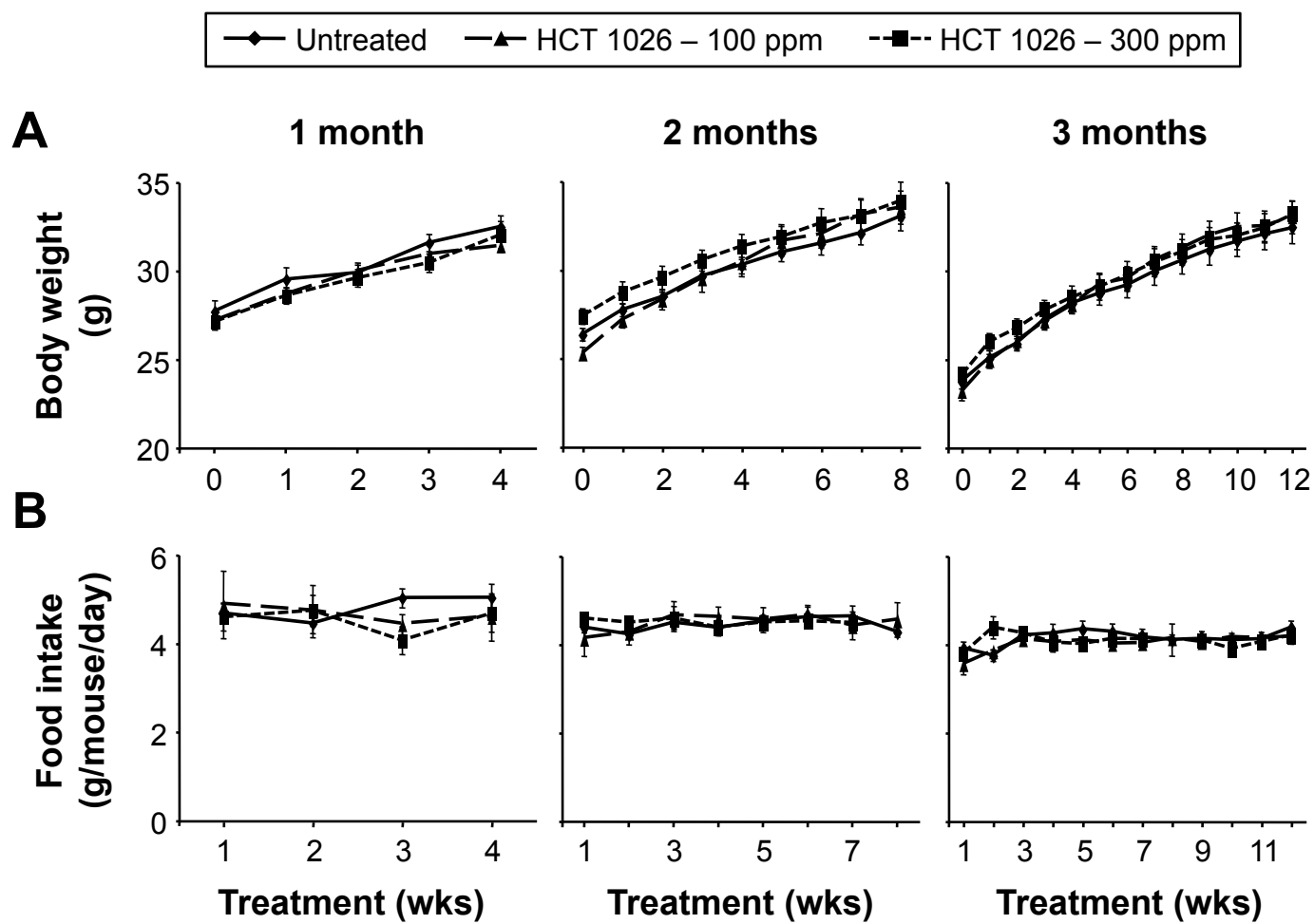

Supplement: Figure S1 — Effect of HCT 1026 treatment on growth and food intake. HCT 1026 was well tolerated by mdx mice with no adverse effects on (A) growth or (B) food intake in treated compared to untreated groups. One-month treatment, n = 13–17 mice per group; two-month treatment, n = 7–15 mice per group; three-month treatment, n = 7–14 mice per group. (PDF) [file pone.0049350.s001.pdf]
